# Factors associated with support for smoke-free policies among government workers in Six Chinese cities: a cross-sectional study

**DOI:** 10.1186/1471-2458-14-1130

**Published:** 2014-11-04

**Authors:** Michelle C Kegler, Xinwei Hua, Madeleine Solomon, Yiqun Wu, Pin Pin Zheng, Michael Eriksen

**Affiliations:** Rollins School of Public Health, Emory University, 1518 Clifton Road NE, 30322 Atlanta, GA USA; Emory Global Health Institute, Emory University, 1599 Clifton Road, 30322 Atlanta, GA USA; Tobacco Technical Assistance Consortium (TTAC), Rollins School of Public Health, Emory University, 1518 Clifton Road NE, 30322 Atlanta, GA USA; ThinkTank Research Center for Health Development, Yizhuang Economic Technological Development Area (BDA), Building 6, Suite 101, Tian Bao Yuan Liu Li, Beijing, 100176 China; Fudan University, 138 Yixueyuan Road, Shanghai, 200032 China; School of Public Health, Georgia State University, 140 Decatur Street, 30303 Atlanta, GA USA

**Keywords:** Tobacco Control, China, Smoke-free policies

## Abstract

**Background:**

A certain level of public support for smoke-free environments is a prerequisite for adoption and enforcement of policies and can be used as an indicator of readiness for legislative action. This study assessed support for comprehensive smoke-free policies in a range of settings such as hotels and colleges among government workers in China and identified factors associated with support for smoke-free policies. Understanding the extent to which government workers, a large segment of the working population in China, report a smoke-free workplace and support for smoke-free policies may be important indicators of readiness for strengthened policies given their role in formulating, implementing and enforcing regulations.

**Methods:**

Data were from an evaluation of the Tobacco Free Cities initiative of Emory University’s Global Health Institute-China Tobacco Control Partnership. Self-administered surveys were completed by 6,646 workers in 160 government agencies in six Chinese cities. Multivariate logistic regression was used to identify factors associated with support for smoke-free worksites, bars, hotels, and colleges.

**Results:**

Over half (54.6%) of participants were male. A large percentage of the male workers smoked (45.9%,) whereas very few women did (1.9%). Fewer than 50% of government workers reported smoke-free policies at work, with 19.0% reporting that smoking is allowed anywhere. Support for smoke-free policies was generally very high, with the lowest levels of support for smoke-free bars (79.0%) and hotels (82.3%), higher levels of support for restaurants (90.0%) and worksites (93.0%), and above 95% support for hospitals, schools, colleges, public transportation and religious settings. Knowledge of the harmfulness of secondhand smoke was positively associated with support for smoke-free policies. Stricter worksite smoking policies were associated with support for smoke-free workplaces and bars, but not hotels and colleges. Women and nonsmokers were more supportive of smoke-free policies in general.

**Conclusion:**

Government workers play important roles in formulating, implementing and enforcing regulations; results suggest support for a more comprehensive approach to smoke-free environments in China among workers across a broad range of agencies.

## Background

With its high prevalence of smoking among men (52.9% in 2010) and modest regulations and enforcement of smoke-free environments, secondhand smoke (SHS) is a major public health problem in China [1–4]. Data from the 2010 Global Adult Tobacco Survey documented that 52.5% of adult nonsmokers were exposed to SHS daily in China and 72.4% were exposed in a typical week [4]. Exposure to SHS causes more than 20,000 lung cancer deaths a year in China and more than 30,000 deaths from ischemic heart disease per year [5]. This burden is disproportionately borne by women and does not include the toll exposure to SHS takes on children [4, 5]. The economic burden of SHS is also considerable [6, 7].

One of the key provisions of the Framework Convention on Tobacco Control is to protect the public from exposure to SHS [8]. Like many countries, the Chinese government has not yet instituted a comprehensive smoke-free air law that covers all worksites and public places, although it has issued a few sector-specific regulations and recently directed high level government officials not to smoke in public [1, 4, 9, 10]. The national regulation for smoke-free health care facilities, which went into effect in 2011, requires smoke-free indoor environments, visible non-smoking signs and complete enforcement [1, 9]. Implementation and enforcement of these regulations varies widely, however [11]. National guidelines also exist for smoke-free primary and secondary schools, and smoking was prohibited at primary and middle schools in 2014 [12]. Additionally, progress is underway in creating smoke-free legislation at the city level, although enforcement issues remain [13–15].

A certain level of public support for smoke-free environments is a prerequisite for adoption and enforcement of policies and can be used as an indicator of readiness for legislative action [16–20]. Prior research shows that working in a smoke-free environment is associated with higher levels of support for smoke-free policies in a range of countries [21–24]. In the U.S. and other developed countries, workplaces, including government buildings, were some of the first places to go smoke-free [19, 25]. It is unclear whether this is the case in China as well.

Belief in the harmfulness of SHS is also associated with support for public and private smoke-free policies in China and elsewhere [17, 22, 26, 27]. In a survey of the general public in six Chinese cities, Li et al. reported that support for smoke-free policies in workplaces and restaurants/bars was associated with knowledge about the dangers of SHS [22]. Interestingly, the Global Adult Tobacco Survey conducted in China in 2010 found that knowledge about the harmfulness of SHS varied by occupation [28]. For example, 62.3% of health care professionals believed that SHS causes heart disease in adults, lung disease in children and lung disease in adults, compared to only 38.8% of organizational leaders and 14.9% of agricultural workers [22]. Beliefs of government workers with respect to the negative effects of SHS or support for smoke-free policies have not been previously studied in China.

The purpose of the current study is to examine support for smoke-free settings among government workers from 160 agencies in six Chinese cities. Nine settings are examined, including colleges, hotels and religious sites which have not been examined in prior studies.

Understanding the extent to which government workers, a large segment of the working population in China, report a smoke-free workplace, their knowledge about the harmfulness of SHS, and whether these factors influence support for smoke-free policies may be important indicators of readiness for strengthened regulations given the role of government workers in formulating and/or enforcing regulations.

## Methods

This study is a secondary analysis of evaluation data from the Tobacco-free Cities initiative of Emory University’s Global Health Institute-China Tobacco Control Partnership [13]. This initiative funded 17 cities for up to 3.5 years to implement action plans largely directed at creating smoke-free environments. The six cities included in the current paper selected smoke-free government agencies as one of their intervention targets.

### Study participants

Study participants were 6,646 employees from 160 government agencies targeted for intervention by city-level grantees (Table 1). Of the 6,646 who completed the survey, 5,405 had complete data on key variables of interest and are included in the analyses reported here. Those excluded from analyses did not differ significantly on any of the demographic variables reported in Table 2. A broad range of government agencies were included, such as Departments of Education, Health Bureaus, and the Office of Chinese Peasants & Workers Democratic Party. The most common types of agencies can be classified as economic/finance, policy, human resources/social services and districts. Health departments, transportation, environment/natural resources, public safety/security, education, research, and information technology agencies were also included in the sample. At the time of data collection in 2011, none of the cities had city-level smoke-free policies and all were covered by the national regulations for smoke-free health care facilities and national guidelines for schools, although neither was fully implemented yet.

**Table 1:** **Description of sites and respondents by city**

City	City population*	Province	Number of sites**	Number of respondents	Number included for analysis
Changchun	2,750,204	Jilin	8	1638	1233
Kelamayi	391,008	Xinjiang	37	844	482
Nanning	6,661,600	Guangxi	13	427	313
Suzhou	10,465,994	Jiangsu	29	1432	1100
Tangshan	7,577,284	Hebei	9	920	894
Yinchuan	1,993,088	Ningxia	64	1385	1383
**Total**	**29,839,178**		**160**	**6,646**	**5,405**

*City population is based on 2010 China National Census Data. **Some sites include multiple agencies.

**Table 2:** **Participant characteristics by smoking status**

Characteristics	Total, N (%) N = 5405	Smokers (N = 1400)	Non-smokers (N = 4005)	p value*
**Gender**				<0.0001
Male	2951 (54.6)	1354 (96.7)	1597 (39.9)	
Female	2454 (45.4)	46 (3.3)	2408 (60.1)	
**Age (Years)**				<0.0001
17-30	1455 (26.9)	252 (18.0)	1203 (30.0)	
31-40	1929 (35.7)	473 (33.8)	1456 (36.4)	
41 or above	2021 (37.4)	675 (48.2)	1346 (33.6)	
**Ethnicity**				0.7605
Han	4892 (90.5)	1270 (90.7)	3622 (90.4)	
Others	513 (9.5)	130 (9.3)	383 (9.6)	
**Education**				<0.0001
Less than or High school completed	1157 (21.4)	382 (27.3)	775 (19.4)	
College/University completed	3743 (69.3)	915 (65.4)	2828 (70.6)	
Post graduate degree completed	505 (9.3)	103 (7.4)	402 (10.0)	
**Length of employment with agency**				<0.0001
Less than 2 years	1292 (23.9)	263 (18.8)	1029 (25.7)	
2-5 years	942 (17.4)	255 (18.2)	687 (17.2)	
More than 5 years	3171 (58.7)	882 (63.0)	2289 (57.2)	
**Self-reported smoking policy at work**				<0.0001
Smoking is allowed anywhere/There is no policy	1028 (19.0)	259 (18.5)	769 (19.2)	
Smoking is allowed only in some indoor areas	1375 (25.4)	521 (37.2)	854 (21.3)	
Smoking is not allowed in any indoor areas	2567 (47.5)	561 (40.1)	2006 (50.1)	
Don’t know	435 (8.1)	59 (4.2)	376 (9.4)	
**Knowledge about dangers of SHS**				
SHS causes serious illness among non-smokers	4931 (91.2)	1147 (81.9)	3784 (94.5)	<0.0001
SHS causes heart disease in adults	3699 (68.4)	884 (63.1)	2815 (70.3)	<0.0001
SHS causes lung disease in children	4621 (85.5)	1079 (77.1)	3542 (88.4)	<0.0001
SHS causes lung cancer in adults	4721 (87.4)	1091 (77.9)	3630 (90.6)	<0.0001

*Chi-square test.

### Data collection procedures

Data were collected by local grantees, which were most commonly city-level Chinese Centers for Disease Control (CDCs) and Health Education Institutes. Recruitment methods were determined by local grantees. Although stratified cluster sampling with random selection of employees was encouraged, sites varied in their recruitment approach. Study protocols were reviewed and approved by local Institutional Review Boards (IRB) in China, including the Jilin Provincial Medical Association IRB for Changchun, the Kelamayi Municipal Medical Association IRB, the Guangxi Provincial Health Bureau IRB for Nanning, the First Affiliated Hospital IRB of Suzhou University, the North China Coal Medical School Affiliated Hospital IRB for Tangshan, and the Yinchuan Municipal Medical Association IRB. Data were collected using self-administered surveys developed by the Emory team that provided technical assistance and training to the cities on evaluation. Surveys were developed for each sector targeted by the grantees, with a core set of common measures across all survey tools. Measures reported here were from the China Global Adult Tobacco Survey (GATS) [2, 29]. The Emory team trained grantees on evaluation methods, and cities were responsible for recruitment, data collection and data entry using a standardized database. Grantees provided Emory with de-identified data sets which were then pooled for the analyses reported here.

### Measures

The dependent variable, support for smoke-free policies, was assessed by asking: Do you think smoking should be permitted in the interior spaces of the following types of places: hospitals, work settings, restaurants, bars, elementary and middle schools, colleges, public transportation (e.g., airports, train stations, buses), religious sites and hotels [29]. Response options were: should be permitted and should not be permitted. The worksite policy question, from GATS, asked: “Which of the following best describes the indoor smoking policy where you work?” Response options included: smoking is allowed anywhere, smoking is allowed only in some indoor areas, smoking is not allowed in any indoor areas, there is no policy, don’t know. Beliefs about harms of SHS were also assessed using items from GATS. Demographics variables included gender, age, ethnicity, education, and length of time working for current employer.

### Statistical analysis

For each of the dependent variables of interest, we conducted unadjusted analyses to assess univariate associations with each of the major independent variables and demographic characteristics. We used multivariate logistic regression to identify factors associated with support for policies in four settings with lower levels of support and/or high potential for tobacco control initiatives: worksites, bars, hotels and colleges. Interaction between smoking status, gender and each explanatory variable was assessed using backwards elimination from full models. In the final multivariable logistic regression models, we accounted for clustering within sites through use of Generalized Estimating Equations (GEEs). We used PROC GENMOD in SAS Version 9.3 (SAS Institute, Cary, NC) to fit the models.

## Results

### Description of study participants

Overall, 54.6% of the participants were men and 25.9% were current smokers (1.9% of women and 45.9% of men) (Table 2). The majority had worked for their agency over five years (58.7%). Of note, less than half (47.5%) reported that smoking was not allowed in any indoor areas at their worksite. Knowledge of the harms of SHS exposure ranged from 68.4% believing it causes heart disease in adults to 91.2% believing it causes serious illness among nonsmokers. Smokers were largely men (96.7%), older and more likely to have a high school education or less. Relative to nonsmokers, smokers were significantly less likely to report a smoke-free worksite and less likely to believe SHS was harmful to nonsmokers.

### Support for smoke-free settings

Table 3 shows support for smoke-free policies in a range of settings. Support was highest for smoke-free elementary and middle schools (97.8%), smoke-free hospitals (96.7%), smoke-free religious sites (96.9%), and smoke-free colleges (97.1%). Support was lower for smoke-free bars (79.0%), smoke-free hotels (82.3%) and smoke-free restaurants (90.0%). Smokers were significantly less supportive of smoke-free policies than nonsmokers across all settings. Smokers were particularly non-supportive of smoke-free policies in bars (54.4%) and hotels (56.6%).

**Table 3:** **Support for smoke-free settings among government workers in six Chinese cities, by smoking status**

Variables	Total (N =5405)	Smokers (N =1400)	Non-smokers (N =4005)	p value*
Hospitals	5228 (96.7)	1292 (92.3)	3936 (98.3)	<0.0001
Workplaces	5029 (93.0)	1102 (78.7)	3927 (98.1)	<0.0001
Restaurants	4865 (90.0)	1016 (72.6)	3849 (96.1)	<0.0001
Bars	4272 (79.0)	762 (54.4)	3510 (87.6)	<0.0001
Elementary and middle schools	5286 (97.8)	1338 (95.6)	3948 (98.6)	<0.0001
Colleges	5246 (97.1)	1305 (93.2)	3941 (98.4)	<0.0001
Public transportations	5189 (96.0)	1276 (91.1)	3913 (97.7)	<0.0001
Religious sites	5235 (96.9)	1302 (93.0)	3933 (98.2)	<0.0001
Hotels	4446 (82.3)	792 (56.6)	3654 (91.2)	<0.0001

*Chi-square test.

### Multivariate associations with support for smoke-free settings

#### Support for smoke-free worksites

Table 4 shows factors associated with support for smoke-free worksites. Nonsmokers were significantly more supportive of smoke-free worksites than were smokers (OR = 7.17, CI = 5.02, 10.24). Workers with the greatest knowledge of the harms of SHS (OR 3.69, CI = 2.27, 5.98) were also more supportive of smoke-free worksites, as were those who reported they worked in a smoke-free environment (OR = 2.46, CI = 1.73, 3.52). Additionally, women were more supportive than men, those with a graduate degree were more supportive than those with a high school education or less, and older workers were less supportive of smoke-free worksites than younger workers.

**Table 4:** **Multivariate logistic regression models for support of smoke-free policies in worksites, bars, and hotels**

	Worksites	Bars	Hotels
	OR (95% CI)	OR (95% CI)	OR (95% CI)
**Smoking status**			
Smoker	Ref	Ref	Ref
Non-smoker	7.17 (5.02, 10.24)	6.00 (4.82, 7.48)	6.68 (5.46, 8.19)
**Self-reported smoking policy at work**			
Smoking is allowed anywhere/There is no policy	Ref	Ref	Ref
Smoking is allowed only in some indoor areas	1.06 (0.74, 1.52)	0.84 (0.66, 1.06)	0.70 (0.52, 0.93)
Smoking is not allowed in any indoor areas	2.46 (1.73, 3.52)	1.43 (1.13, 1.82)	1.17 (0.89, 1.55)
Don’t know	1.13 (0.68, 1.89)	1.12 (0.86, 1.46)	0.79 (0.56, 1.13)
**Gender**			
Male	Ref	Ref	Ref
Female	2.39 (1.65, 3.46)	0.98 (0.80, 1.20)	1.29 (1.04, 1.61)
**Age (Years)**			
17-30	Ref	Ref	Ref
31-40	0.64 (0.45, 0.92)	1.39 (1.13, 1.72)	1.27 (1.01, 1.61)
41 or above	0.69 (0.51, 0.95)	1.83 (1.51, 2.23)	1.53 (1.24, 1.90)
**Education**			
Less than or High school completed	Ref	Ref	Ref
College/University completed	1.09 (0.77, 1.55)	1.10 (0.90, 1.35)	1.04 (0.82, 1.32)
Post graduate degree completed	2.29 (1.36, 3.84)	1.10 (0.82, 1.49)	1.13 (0.82, 1.57)
**Length of employment with Agency**			
Less than two years	Ref	Ref	Ref
2-5 years	1.19 (0.77, 1.83)	1.15 (0.93, 1.43)	1.23 (0.97, 1.57)
More than 5 years	1.05 (0.71, 1.54)	1.43 (1.17, 1.75)	1.18 (0.94, 1.47)
**Knowledge of SHS hazards**			
Score = 0	Ref	Ref	Ref
Score = 1	1.74 (0.95, 3.18)	1.18 (0.80, 1.75)	0.96 (0.64, 1.45)
Score = 2	1.83 (1.00, 3.33)	1.06 (0.73, 1.56)	1.00 (0.65, 1.55)
Score = 3	2.61 (1.48, 4.58)	1.33 (0.94, 1.87)	1.08 (0.72, 1.63)
Score = 4	3.69 (2.27, 5.98)	2.64 (1.93, 3.59)	2.48 (1.68, 3.67)

#### Support for smoke-free bars

Nonsmokers were more supportive of smoke free policies in bars compared to smokers (OR = 6.00, 95% CI: 4.82, 7.48). Those with a stricter worksite policy were more supportive of smoke-free bars (OR = 1.43, CI = 1.13, 1.82). Similarly, those with the highest level of knowledge about SHS were more likely to support smoke-free bars (OR = 2.64, CI = 1.93, 3.59). Lastly, older workers were more supportive of smoke-free bars, as were those who had worked for the agency for a longer period of time.

#### Support for smoke-free hotels

Table 4 also shows factors associated with support for smoke-free hotels. Nonsmokers were significantly more supportive than were smokers (OR = 6.68, CI = 5.46, 8.19). Knowledge of the harmfulness of SHS was associated with support for smoke-free hotels only at the highest level of knowledge (OR = 2.48, CI = 1.68, 3.67). Interestingly, those with partial smoke-free policies in the worksite were less supportive of smoke-free hotels (OR =  .70, CI = 0.52, 0.93) than those with no policy, and stricter policies (i.e., no smoking in any indoor areas) were not associated with support for smoke-free hotels. Women were more likely to support smoke-free hotels, as were older workers.

#### Support for smoke-free colleges

In the model of support for smoke-free colleges, there was a significant interaction between smoking status and age (Table 5). Among smokers, those over 40 were more supportive of smoke-free colleges than were those aged 17–30 (OR = 1.87, CI = 1.02, 3.41). Among nonsmokers, workers 40 and older were less supportive of smoke-free colleges (OR = .23, CI = 0.9, 0.56). Interestingly, worksite policies did not predict support for smoke-free colleges, although knowledge of SHS hazards did at the two higher levels of knowledge. Gender also predicted support (OR = 1.98, CI = 1.21, 3.25) for smoke-free colleges, whereas education and length of work for the agency did not.

**Table 5:** **Multivariate logistic regression models for support of smoke-free policies in colleges**

	Colleges
	OR (95% CI)
**Smoking status**	
Interaction between smoking status and age	
Smoker	
Age 17-30	Ref
Age 31-40	1.66 (0.91, 3.03)
Age 40+	1.87 (1.02, 3.41)
Nonsmoker	
Age 17-30	Ref
Age 31-40	0.26 (0.11, 0.62)
Age 40+	0.23 (0.90, 0.56)
**Self-reported smoking policy at work**	
Smoking is allowed anywhere/There is no policy	Ref
Smoking is allowed only in some indoor areas	0.93 (0.63, 1.38)
Smoking is not allowed in any indoor areas	1.71 (0.97, 3.03)
Don’t know	0.51 (0.21, 1.22)
**Gender**	
Male	Ref
Female	1.98 (1.21, 3.25)
**Education**	
Less than or High school completed	Ref
College/University completed	0.86 (0.59, 1.24)
Post graduate degree completed	1.39 (0.67, 2.89)
**Length of employment at agency**	
Less than two years	Ref
2-5 years	1.19 (0.65, 2.18)
More than 5 years	0.97 (0.60, 1.57)
**Knowledge of SHS hazards**	
Score = 0	Ref
Score = 1	1.43 (0.73, 2.78)
Score = 2	1.75 (0.75, 4.08)
Score = 3	2.92 (1.37, 6.23)
Score = 4	2.45 (1.31, 4.56)

## Discussion

This study is the first to examine support for smoke-free settings among government workers, an important set of opinion leaders and a large segment of the working population in China. It is also the first to examine support for smoke-free hotels and colleges in China and among the first to examine support for smoke-free restaurants, bars, worksites and public transportation. Results suggest that support for a range of smoke-free public places was quite high among government workers participating in this study. Over 90% felt that smoking should not be permitted in hospitals, elementary and middle schools, colleges, public transportation, religious sites, worksites or restaurants. Support was lower, but still quite high, for smoking restrictions in bars and hotels.

Our results suggest higher support for smoke-free environments among government workers than among the general public or perhaps a shift toward greater support in recent years. Data collected in 2006 from six Chinese cities showed that support for bans was over 90% for schools and public transportation vehicles, but was lower for hospitals, worksites, and restaurants and bars among the general public. Measurement issues may contribute to these differences. We asked about interior spaces and only offered two responses (should be/should not be permitted). Other studies examining public support have offered a response option of partial restrictions [20, 22]. We also clearly specified indoor areas which may have also elevated support given that support for smoking restrictions in outdoor places tends to be lower, albeit growing [30]. Additionally, the school-related question asked about elementary and middle schools and not secondary schools. Support may have been lower if high schools had been included in the question. Even with these caveats, support was surprisingly high given the tobacco control context in China, with a majority of men still smoking.

Levels of support for smoke-free public places typically vary by smoking status, knowledge about the harmfulness of SHS, and demographics [18, 20–22, 31, 32]. Levels of support also vary by the type of restriction [30, 33]. Similar to other studies, we found that knowledge about SHS was associated with increased support [22]. Knowledge about the harmfulness of secondhand smoke exposure is lower in China than in many other countries [22, 28]. In the current study, general knowledge about the harms of SHS was over 90%, but knowledge on the specific diseases caused by SHS, particularly heart disease in adults, was lower.

The current study also examined the hypothesis that employees who report stricter worksite policies are more supportive of smoke-free places than those who report less restrictive policies. This association was significant for worksites and bars, but not for hotels and college campuses. It may be that experience with smoking restrictions at work do not generalize to support for smoke-free places that include a residential aspect. Other studies have examined the impact of worksite and/or comprehensive smoke-free policies for public places on support for smoke-free worksites, restaurants, bars, trains/train stations and homes and found positive associations [21–23, 34, 35]. We are not aware of any other studies, however, that have examined the influence of a worksite policy on support for the full range of settings examined in our study.

Numerous prior studies have documented that smokers are less supportive of smoke-free policies [17, 20, 22]. As expected, our study documented that smoking status was associated with support for smoke-free worksites, hotels and bars. Associations between smoking status and support for smoke-free colleges were more complex, with interactions observed between age and smoking status. Older smokers were more supportive of smoke-free colleges than were younger smokers, perhaps because it was a more salient issue for younger smokers. Older nonsmokers, in contrast, were less supportive of smoke-free colleges than were younger nonsmokers. Again, this may be due to the personal relevance of the issue for younger nonsmokers who more easily recalled being bothered by SHS on campus. Another expected finding was that women were generally more supportive of smoke-free policies than men, even when smoking status was controlled [17, 20, 22]. In our study, women were more supportive of smoke-free colleges, worksites and hotels than were men.

This study has several limitations. The first is related to generalizability of the findings. The selected agencies covered a broad range of agency types with relatively few health agencies included. The government agencies targeted by the grantees, however, may not be representative of all government agencies in the participating cities. Generalizability of the results is further limited by the likely convenience sampling of workers in at least some of the agencies and our inability to calculate a response rate. It is possible that workers that were more supportive of smoke-free environments were more likely to complete the survey. However, we did have a high prevalence of smoking among male workers, thus suggesting limited bias by smoking status. Secondly, we assessed perception of the worksite policies rather than the actual policy. This approach, while perhaps misclassifying actual policies, does capture differences in enforcement or simply differences in awareness of the existing policy. A strong social desirability bias may have also existed. Lastly, we were unable to distinguish the type of government agency by individual respondent, thereby limiting our ability to seek differences in support by government agency function (e.g., health versus commerce).

## Conclusion

This study has implications for tobacco control in China. First, as suggested by prior studies, those who know the dangers of SHS are more likely to support smoke-free policies [17, 22, 26, 27]. Given lower levels of knowledge about SHS in China relative to elsewhere, a public education campaign focused on SHS would likely build support for smoke-free policies. Second, the low support for smoke-free hospitality venues may reflect the misconception among government workers that smoke free policies may have a negative economic impact. Disseminating international experience to the contrary may help to dispel such misunderstandings. Based on the U.S. experience, smoke-free policies often start locally with an emphasis on worksites [19, 25]. Other countries have taken a more top down approach [19]. Although it is not clear which approach would work best in China, our study suggests that China may have support from an important group of opinion leaders for further adoption of smoke-free environments across a range of settings.
